# Mutant Alleles of *Photoperiod-1* in Wheat (*Triticum aestivum* L.) That Confer a Late Flowering Phenotype in Long Days

**DOI:** 10.1371/journal.pone.0079459

**Published:** 2013-11-14

**Authors:** Lindsay M. Shaw, Adrian S. Turner, Laurence Herry, Simon Griffiths, David A. Laurie

**Affiliations:** Department of Crop Genetics, John Innes Centre, Norwich, United Kingdom; Ohio State University, United States of America

## Abstract

Flowering time in wheat and barley is known to be modified by mutations in the *Photoperiod-1* (*Ppd-1*) gene. Semi-dominant *Ppd-1a* mutations conferring an early flowering phenotype are well documented in wheat but gene sequencing has also identified candidate loss of function mutations for *Ppd-A1* and *Ppd-D1*. By analogy to the recessive *ppd-H1* mutation in barley, loss of function mutations in wheat are predicted to delay flowering under long day conditions. To test this experimentally, introgression lines were developed in the spring wheat variety ‘Paragon’. Plants lacking a *Ppd-B1* gene were identified from a gamma irradiated ‘Paragon’ population. These were crossed with the other introgression lines to generate plants with candidate loss of function mutations on one, two or three genomes.

Lines lacking *Ppd-B1* flowered 10 to 15 days later than controls under long days. Candidate loss of function *Ppd-A1* alleles delayed flowering by 1 to 5 days while candidate loss of function *Ppd-D1* alleles did not affect flowering time. Loss of *Ppd-A1* gave an enhanced effect, and loss of *Ppd-D1* became detectable in lines where *Ppd-B1* was absent, indicating effects may be buffered by functional *Ppd-1* alleles on other genomes. Expression analysis revealed that delayed flowering was associated with reduced expression of the *TaFT1* gene and increased expression of *TaCO1*.

A survey of the GEDIFLUX wheat collection grown in the UK and North Western Europe between the 1940s and 1980s and the A.E. Watkins global collection of landraces from the 1920s and 1930s showed that the identified candidate loss of function mutations for *Ppd-D1* were common and widespread, while the identified candidate *Ppd-A1* loss of function mutation was rare in countries around the Mediterranean and in the Far East but was common in North Western Europe. This may reflect a possible benefit of the latter in northern locations.

## Introduction

Wheat (*Triticum* species) and barley (*Hordeum vulgare*) in their ancestral forms are quantitative long day plants. That is, they are stimulated to flower by increasing day length [1]. In barley, a recessive mutation at the *Photoperiod-H1* (*Ppd-H1*) locus allele attenuates the long day photoperiod response to confer a later flowering phenotype [2], [3]. This has a yield benefit in environments with long growing seasons such as North Western Europe [2].

Colinear with the *Ppd-H1* gene on barley chromosome 2H are a homoeologous series of *Ppd-1* genes on the group 2 chromosomes of durum and bread wheat (*T. turgidum ssp. durum , T. aestivum*). In contrast to barley, the previously characterized mutations in wheat are partially dominant and confer an early flowering phenotype in short day (SD) or long day (LD) conditions. This day neutral or “photoperiod insensitive” (PI) phenotype is widely used in environments where the optimal growing conditions occur under SD conditions or where early flowering is desirable to avoid summer temperature and drought stresses [4]–[6]. PI is associated with promoter deletions [7]–[9], a transposon insertion in the promoter of *Ppd-1* [9] or increased gene copy number of *Ppd-1* [10]. PI alleles are designated by an *a* suffix (*Ppd-1a*) while alleles conferring a photoperiod sensitive phenotype have a *b* suffix (*Ppd-1b* [11]).

The expression of *Ppd-1b* alleles follows a diurnal cycle with very low expression at dawn followed by a morning peak in expression which falls to very low levels at night. Early flowering PI (*Ppd-1a*) alleles lose this diurnal pattern and have constantly elevated expression which is closely associated with the increased expression of *TaFT1* (also called *VRN-3*), a wheat orthologue of the Arabidopsis *FT* gene [7], [8], [12], [13]. In contrast, the late flowering *ppd-H1* mutation in barley is associated with reduced expression of *HvFT1* [3], [14]. In Arabidopsis, FT protein is a mobile signal produced in leaves which moves to the shoot apex where it interacts with the FD protein to induce flowering by promoting the expression of *APETALA1* [15]–[19]. The cereal orthologues are predicted to act in a similar manner [20].

*Ppd-1* is a member of the pseudo-response regulator (*PRR*) gene family. PRR proteins have two conserved domains. The first is the pseudo-receiver domain near the N terminus. The second is a 43 amino acid region near the C terminus, termed the CCT domain, which also contains the nuclear localization signal for the protein [21]. Proteins containing a CCT domain act as transcription factors, either in complexes with NF-YC (also known as HAP) proteins [22]–[24] or by direct binding to DNA [25].

Four candidate reduced or loss of function alleles of *Ppd-1* were previously identified by sequencing. The *Ppd-A1* allele in ‘Paragon’ has a 39 bp deletion at the transcription start site associated with low expression [13]. The exons are intact, so this allele is likely to produce a reduced level of functional protein. We refer to this as the *Ppd-A1*_promdel allele here. The *Ppd-A1* allele from ‘Cappelle-Desprez’ has a 303 bp deletion that removes parts of exons 5 and 6 and intron 5, producing a frameshift mutation predicted to give a truncated protein lacking the CCT domain [7]. ‘Norstar’ was subsequently found to carry the same mutation. We refer to this as the *Ppd-A1*_delCN allele here. The *Ppd-D1* allele from ‘Norstar’ has a 5 bp deletion in exon 7 that gives a frameshift mutation predicted to produce a truncated protein lacking the CCT domain [7]. We refer to this as the *Ppd-D1*_delN allele here. The *Ppd-D1* allele found in ‘Mercia’ and subsequently in ‘Paragon’ has a 4.8 kb *Mariner* type transposable element inserted into intron 1 [7]. A splice site within the *Mariner* element produces a transcript with a premature stop codon and a predicted protein comprising only the N-terminus and part of the pseudo-receiver domain [13]. We refer to this as the *Ppd-D1*_Mar allele here. From work described above it is highly unlikely that PRR proteins lacking a CCT domain can function correctly, suggesting that *Ppd-A1*_delCN and *Ppd-D1*_delN are loss of function or reduced function alleles. *Ppd-D1*_Mar is predicted to be non-functional, or weakly functional if some correctly spliced transcript is produced.

Previous results from barley show that a recessive *ppd-H1* mutation extends the life cycle of the plant under LD's [2], [3]. This suggested that wheat varieties bred for environments with long growing seasons, such as those in NW Europe, might resemble barley in having mutations that reduce *Ppd-1* activity and delay flowering in long day conditions. Alternatively, weak or loss of function *Ppd-1* alleles in wheat might be phenotypically neutral if functional versions are present on one or more of the other genomes.

Guo et al. [26] analyzed *Ppd-D1* alleles in 492 modern hexaploid wheat accessions including 216 from China. They found that the *Ppd-D1*_delN and *Ppd-D1*_Mar alleles (their haplotypes IV and III, respectively) were widely distributed globally but with the exception of China the number of accessions recorded per country was too low to test association with environment. The phenotypic effect of candidate loss of function alleles in wheat is therefore unclear and needs to be tested experimentally. We assessed their flowering time phenotypes using a series of introgression lines developed in the photoperiod sensitive hexaploid spring variety ‘Paragon’. Crossing introgression lines with ‘Paragon’ gamma ray mutants lacking the *Ppd-B1* gene enabled the development of wheat plants with candidate loss of function alleles on one, two or three genomes. Measurements of flowering time were combined with analyses of gene expression to assess the relationship between flowering phenotype and the expression of *TaFT1* and *TaCO1*. This provided a direct comparison with the effects of PI early flowering *Ppd-1a* alleles in a ‘Paragon’ background [10], [13], [27].

The candidate loss of function alleles we used were identified by gene sequencing using a small number of genotypes with defined photoperiod responses [7]. To determine if these alleles were common or rare we also genotyped two contrasting germplasm collections for the PI *Ppd-D1a* allele and the *Ppd-A1*_delCN, *Ppd-D1*_delN and *Ppd-D1*_Mar alleles. From these results we aim to identify the phenotypic effects of candidate loss of function *Ppd-1* alleles in wheat and their distribution.

## Materials and Methods

### Diagnostic assays

Alleles were identified using gel-based assays based on size differences in PCR product and by fluorescent assays with the KASPar system (KBiosciences, UK; http://www.kbioscience.co.uk).

#### *Ppd-A1*_promdel

This small deletion occurs in a GC rich region and we were unable to develop a reliable assay. Therefore, this allele was not assessed in the germplasm collections.

#### *Ppd-A1*_delCN

Gel-based assay: Primary amplification; AgF3 (agtcagagatatgcagcaac) and HvR6-1 (tcttcccgaagttcctctc). PCR conditions; 94°C for 2 min, 30 cycles of [94°C for 20 sec; 55°C for 20 sec; 72°C for 90 sec]. Secondary amplification; AgF3 and 219-R2 (tgccgttgattggcgagac). PCR conditions as for primary amplification. The intact (884 bp) and deletion (581 bp) products were resolved using 10 µl of PCR product in 1.2% agarose gels in TAE buffer. KASPar assay: Common primer Cdex5-6IDL (cctgaagtcagagatatgcagcaac), Intact allele Cdex5-6IDI (GAAGGTGACCAAGTTCATGCTcattagtttcttttggtttctggca), Deletion allele Cdex5-6IDD (GAAGGTCGGAGTCAACGGATTcaatcagatcagcagctcgaac). PCR conditions; 94°C for 15 min, 20 cycles of [94°C for 10 sec; 57°C for 5 sec; 72°C for 10 sec], 24 cycles of [94°C for 10 sec, 57°C for 20 sec, 72°C for 40 sec]. Fluorescence end point reading was taken at 25°C.

#### *Ppd-D1*_delN

Gel-based assay: PpdD1_Nordel_F4_M13 ( tgtaaaacgacggccagtgtctccaatcaaggcggt (M13 tail underlined) and PpdD1_NordelR3 (gggcgaaaccttattatttccg) plus labelled M13 primer. PCR conditions; 95°C for 15 min, 40 cycles of [94°C for 40 sec; 55°C for 30 sec; 72°C for 40 sec]. The intact (189 bp) and deletion (184 bp) products were separated by capillary electrophoresis. KASPar assay: Common primer TaPpdDD002FL (ggtctccaatcaaggcggt), Intact allele TaPpdDD002RI (GAAGGTGACCAAGTTCATGCTcgagcagctcccgacg), Deletion allele TaPpdDD002RD (GAAGGTCGGAGTCAACGGATTgggcgagcagctccaac). PCR conditions were the same as for *Ppd-A1*_delCN.

#### *Ppd-D1*_Mar

Gel-based assay: 2D_Mar_F1 (acggactactcctccatcg), 2D_Mar_F2 (ggcaccatttgacaggcag) and 2D_Mar_R1 (cgggaggaagatttggac) were combined in one reaction. PCR conditions; 95°C for 15 min, 40 cycles of [94°C for 40 sec; 55°C for 30 sec; 72°C for 90 sec]. The transposon absent (1232 bp) and transposon present (727 bp) products were resolved using 10 µl of PCR product in 1.2% agarose gels in TAE buffer.

KASPar assay: Common primer TaPpdDI001FL (tgttaattaatttgtactggctcggta), Insertion present TaPpdDI001RI (GAAGGTCGGAGTCAACGGATTtgacttatacacccggacggag), Insertion absent TaPpdDI001RD (GAAGGTGACCAAGTTCATGCTgaacatgacacacaaccaacgc). PCR conditions were the same as for *Ppd-A1*_delCN.

#### *Ppd-B1* deletions

These were selected by PCR using primers BgF1 (agacgattcattccgctcc) and HvR6-2 (agcagcaccatttgagagg). *Ppd-B1* hemizygotes were selected during backcrossing using the TaqMan® assay described in [10].

### Plant material

Introgression lines for candidate loss of function *Ppd-1* alleles were developed in the photoperiod sensitive hexaploid spring wheat variety ‘Paragon’ by recurrent backcrossing and selection with the markers described above. For *Ppd-A1*_delCN, independent lines were developed using ‘Cappelle-Desprez’ or ‘Norstar’ as donors. ‘Norstar’ also provided the *Ppd-D1*_delN deletion. Two introgression lines were developed from each source starting with independent F_1_ plants. Back cross (BC) lines were also developed with *Ppd-A1*_delCN and *Ppd-D1*_delN in combination. *Vrn-1* alleles from the various donor varieties were selected against using published assays [28], [29]. Plants were developed to BC_4_ at which point they were allowed to self-pollinate to produce homozygous BC_4_F_2_ plants and BC_4_F_3_ and F_4_ families.

No candidate loss of function allele for *Ppd-B1* was found in previous investigations so for this study we screened a gamma irradiated ‘Paragon’ population. Dry seeds were exposed to 250 Gy gamma rays and surviving plants were self-pollinated to produce 576 independent M3 lines. Screening using a PCR assay for *Ppd-B1* (above) and subsequent verification with additional primer pairs identified two lines completely lacking the *Ppd-B1* gene (*Ppd-B1*_del211a and *Ppd-B1*_del319c). Previous sequencing of barley BACs identified three genes flanking *Ppd-H1* (*APX1* and *UNK2* proximal and *STK* distal; [3]. PCR assays showed that *Ppd-B1*_del211a and _del319c lacked the three flanking genes. The deletions are therefore larger than the *Ppd-B1* gene, assuming colinearity with barley. For comparison with the B genome deletions, the gamma irradiated population was used to select one line with a complete deletion of *Ppd-A1* (*Ppd-A1*_del128c) and one with a complete deletion of *Ppd-D1* (*Ppd-D1*_del143a) using additional primers AgF5 (cggtctccaatcaaggcc), HvF2 (gatgaacatgaaacggg), HvR6-1 (tcttcccgaagttcctctc) and DgR2 (aagcgagccgcatatgatg). Genome specificities were confirmed using ‘Chinese Spring’ group 2 nullisomic/tetrasomic lines. Selected deletions were backcrossed twice to ‘Paragon’ to reduce the effect of additional background mutations. PCR assays confirmed that each resulting line lacked the *Ppd-1* gene from only one genome.

At the BC_3_ stage, plants heterozygous for *Ppd-A1*_delCN (‘Norstar’ source) were crossed to plants heterozygous for *Ppd-D1*_delN. Progeny heterozygous at both loci were crossed to BC_2_F_2_ homozygotes for *Ppd-B1*_del211a or *Ppd-B1*_del319c. Plants from these crosses (hemizygous for *Ppd-B1* and heterozygous for *Ppd-A1*_delCN and *Ppd-D1*_delN) were allowed to self-pollinate and 768 progeny seedlings were screened to identify plants with different combinations of introgressed mutations (IM). Plants were selected that were homozygous for ‘Paragon’ alleles on all three genomes (0_IM controls) or were homozygous for introgressed alleles on one, two or three genomes (1_IM, 2_IM and 3_IM, respectively; Table S1). These are referred to as plants from the 3× cross to distinguish them from the primary introgression lines.

Selected plants were grown and allowed to self-pollinate. Families of five to 10 progeny from three or four independent plants of each genotype were grown in a heated glasshouse with supplementary lighting giving a fixed 18 h day (experiment 1_winter 2010/2011) and in a glasshouse with frost protection and natural long days (experiment 2_summer 2011; planted on the 3^rd^ May so that plants received 15 h light at the start of development extending to 17 h during the course of the experiment). For each experiment seeds were imbibed for 2 days in the dark at 4°C, germinated for 4 days and planted in soil. Flowering time was recorded for each plant as the date when the spike had emerged half way from the flag leaf on the main stem. Seed from self-pollinated plants was used for subsequent gene expression studies. Throughout backcrossing and self-pollination the emerging spikes were covered with glassine bags to prevent outcrossing. Experiment_1 also included three ‘Paragon’ introgression lines carrying a PI *Ppd-1a* allele developed as described in [27].

Differences in spikelet number were recorded from the spike on the main stem plus the leading tiller of each plant in Experiment_1. Each visible spikelet and the terminal spikelet was counted irrespective of whether or not they had set seed. For most genotypes spikelet number was sampled from 20 (5 from each of 4 families) or 21 (7 from each of 3 families) plants. For the original gamma-ray induced deletion lines and for segregants carrying the 211a B genome deletion, ten plants from one family were analyzed because of lower seed numbers. Ten plants were analyzed for the Paragon control. The mean spikelet number was determined by combining the main stem with the leading tiller. Means were compared by simple regression.

Gel-based or KASPar assays were used to determine the frequency of the *Ppd-A1*_delCN, *Ppd-D1*_delN, *Ppd-D1*_Mar and *Ppd-D1a* alleles in two contrasting germplasm collections. We genotyped 421 varieties from a collection developed for the European Union ‘Genetic Diversity in Agriculture: Temporal Flux, Sustainable Productivity and Food Security’ (GEDIFLUX) project. The GEDIFLUX collection consists of winter wheat varieties grown in the UK or North Western Europe between the 1940s and 1980s and includes varieties that were significant contributors to current European winter wheat pedigrees. We also genotyped 769 accessions from the A.E. Watkins collection which consists of land races and varieties collected in the 1920s and 1930s. The GEDIFLUX and Watkins collections are maintained and available through the UK Wheat Genetic Improvement Network (http://www.wgin.org.uk).

### Sequencing of cDNA clones

Analysis of ‘Paragon’ *Ppd-D1*_Mar transcripts was described in [13]. *Ppd-A1*_delCN and *Ppd-D1*_delN transcripts were isolated and analyzed by the same method from ‘Paragon’ introgression lines. cDNA was prepared from a pool of samples from 3 and 6 h after dawn (1200 and 1500 samples). *Ppd-A1* products were amplified using primers AgF1 (gacacgattcattcccgcc) and AgR4 (cagctgtctaaatagtattacg). *Ppd-D1* products were amplified using DgF1 (ctcaacagcttgctcttgtg) and DgR2 (aagcgagccgcatatgatg) or DgR11 (ctgatcgactccgcacttg). F and R primers were in the 5′ and 3′ untranslated regions, respectively. Following cloning, PCR products were sequenced using BigDye 3.1 reactions resolved on an ABI 3730 by a commercial provider.

### Sampling of introgression lines for analysis of gene expression over a 24 hour period

The following introgression lines were selected for gene expression analysis based on time to flowering; *Ppd-A1*_delCN+*Ppd-D1*_delN, *Ppd-B1*_del319c+*Ppd-D1*_delN and *Ppd-A1*_delCN+*Ppd-B1*_del319c+*Ppd-D1*_delN. Paragon was included as a control. Plants were grown in a controlled environment room under long day conditions (18 h light, 6 h dark) at 16°C. After 22 days plants were sampled at 3 h intervals over a 24 h period. For each time point three biological replicates were taken per genotype. Each replicate was composed of the above ground biomass of three plants with each plant being from an individual family. Four families were used for each genotype. Samples were immediately frozen in liquid nitrogen.

### Sampling of introgression lines for analysis of gene expression over a 6 week time course

‘Paragon’, the four introgression lines used for the 24 h time course plus the following additional introgression lines were selected for expression analysis over six weeks: *Ppd-A1*_delCN, *Ppd-B1*_del319c, *Ppd-D1*_delN, *Ppd-A1*_delCN+*Ppd-B1*_del319c. An early flowering line ‘Paragon (*Ppd-D1a*)’ was included for comparison. Plants were grown in a controlled environment room under long day conditions (18 h light, 6 h dark) at 16°C. Plants were sampled after 9 h light at one, two, three, four, five and six weeks after planting. For each genotype the progeny from four individuals for two families were sown. Three biological replicates were taken at each time point for each genotype. Each biological replicate was composed of the above ground biomass of four plants consisting of two plants from each of two families. Samples were immediately frozen in liquid nitrogen

### Quantitative RT-PCR analysis

RNA was extracted using the hot phenol RNA extraction method described in [30] and treated with DNAse I (Roche Diagnostics). First strand complementary DNA (cDNA) was synthesised using an oligo (dT) and random primer mix (Invitrogen) using Moloney Murine Leukemia Virus Reverse Transcriptase (M-MLV RT) (Invitrogen) following the manufacturer's instructions. Gene expression levels were quantified by real-time PCR using the LightCycler® 480 sequence detection system (Roche) using LightCycler® 480 SYBR Green I Master (Roche) following the manufacturer's instructions. Primers earlyFTF2 (agagccctcgtccgaccat), earlyFTR2 (gttgtagagctcggcgaagtc) (efficiency 2.06) and TaCO-D1 (cagacaccaattcacttcagc and tccacttccatgtctgcat) (efficiency 2.075) [13] were selected for expression analysis of *TaFT1* and *TaCO1*. RNA polymerase 15 kDa subunit (*TaRP15*) was selected as an internal control reference gene. Primer amplification efficiency and gene expression analysis was calculated as described in [13]. Each reported relative expression value corresponds to three biological replicates with two technical replicates for each biological replicate.

## Results

### Development of introgression lines with candidate loss of function *Ppd-1* alleles

To test the effects of candidate loss of function alleles, two introgression lines were developed, each from an independent F_1_ plant, for each of the A, B and D genome mutations in the hexaploid spring wheat variety ‘Paragon’. ‘Paragon’ carries the *Ppd-A1*_promdel allele associated with low levels of expression and the *Ppd-D1*_Mar allele [13]. It also has a *Ppd-B1* gene with no known mutations and a copy number of one [10]. This composition is sufficient to confer a photoperiod sensitive phenotype (late flowering in short days and rapid flowering in long days).

**Figure 1:**
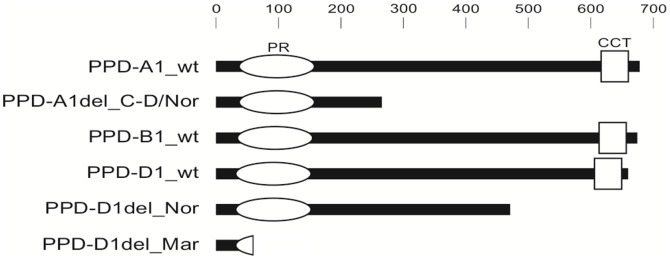
Predicted *Ppd-1* proteins from wild type and candidate loss of function alleles. Pseudo-receiver (PR) and CCT domains are in white. Scale indicates number of amino-acids.

PCR assays were used to introgress *Ppd-A1*_delCN (from ‘Cappelle-Desprez’ and ‘Norstar’ sources) and *Ppd-D1*_delN into ‘Paragon’. Sequencing genomic DNA confirmed that the *Ppd-A1*_delCN alleles from ‘Cappelle-Desprez’ and ‘Norstar’ were identical throughout. Sequencing of *Ppd-D1*_delN was extended upstream from the region described in [7] (DQ885770) but no further mutation was found. Sequenced cDNA clones of *Ppd-A1*_delCN and *Ppd-D1*_delN had frameshift mutations as expected from their respective genomic sequences so that transcripts gave predicted proteins lacking a CCT domain and its associated nuclear localization signal (Fig. 1). These are highly likely to be non-functional.

Previously, we showed that the *Ppd-B1* gene provided approximately 90% of the *Ppd-1* transcript in ‘Paragon’ [13]. As no loss of function mutation had been previously identified for *Ppd-B1* we selected two lines from a 250Gy gamma irradiated ‘Paragon’ population that completely lacked the *Ppd-B1* gene.

### Flowering times of introgression lines with loss of function alleles_Experiment 1

By analogy to the barley *ppd-H1* mutation, loss of function alleles in wheat would be predicted to show a reduced photoperiod response, resulting in a later flowering phenotype in long days. This was tested in two experiments, comparing the 3× cross selections with controls and primary introgression lines.

**Figure 2:**
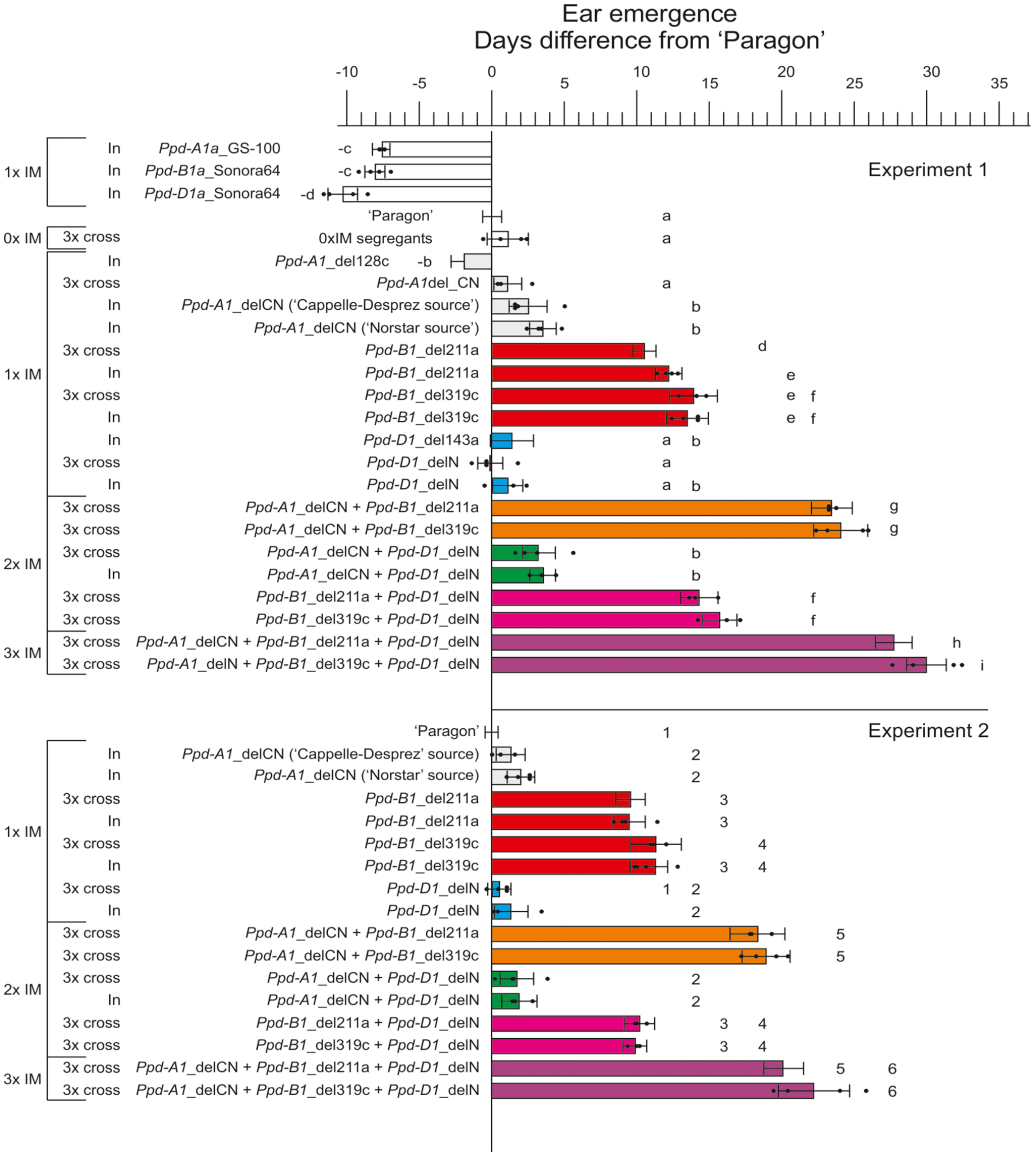
Flowering times (days difference from ‘Paragon’) for homozygous primary introgression lines (In) and homozygous derivatives of the 3× cross with introgressed mutations (IM) on one, two or three genomes. Experiment_1 was carried out in a heated, lit glasshouse with supplementary lighting giving an 18 h day. Experiment_2 was carried out in a glasshouse with natural long days. Black dots show the mean flowering time of individual families within genotypes. For bars without dots only one family was available. The coloured bar and error bar shows the mean and standard deviation of each genotype. Lines with the same lower case letter (Experiment 1) or number (Experiment 2) were not significantly different for flowering time.

Ten ‘Paragon’ control plants and one to four families of five to 10 plants of each genotype were grown in a heated glasshouse with supplementary lighting providing an 18 h light period. Exposure to an 18 h day from the coleoptile stage should maximize phenotypic differences resulting from an impaired photoperiod response under long days. In addition to ‘Paragon’ controls we included families of BC_4_F_4_ plants homozygous for a PI mutation on one genome (‘Paragon (*Ppd-A1a*_GS-100)’; ‘Paragon (*Ppd-B1a*_Sonora64)’ and ‘Paragon (*Ppd-D1a*_Sonora64)’ [27]. ‘Paragon’ control plants reached ear emergence after approximately 62 days. Plants with *Ppd-A1a*, *Ppd-B1a* or *Ppd-D1a* mutations flowered 7, 7 and 10 days earlier, respectively (Fig. 2), consistent with previous studies of the effect of *Ppd-1a* alleles under long days [31], [32].

#### No introgressed mutation (0_IM) genotype

Families were derived from plants that were hemizygous for *Ppd-B1* and heterozygous for *Ppd-A1*_delCN and *Ppd-D1*_delN. This allowed the selection of progeny that were homozygous for ‘Paragon’ *Ppd-1* alleles on all three genomes (0_IM controls). These families had the same flowering time as ‘Paragon’ (Fig. 2), showing that no significant background QTL affecting flowering time under long days were segregating.

#### 1_IM genotypes

Replacement of the endogenous ‘Paragon’ *Ppd-A1*_promdel allele with the *Ppd-A1*_delCN allele gave a small but detectable delay in flowering with families homozygous for the *Ppd-A1*_delCN allele having mean flowering times 1 to 4 days later than ‘Paragon’. This was significant for the introgression (In) BC_4_F_3_ lines from the ‘Cappelle-Desprez’ and ‘Norstar’ sources but not for the 3× cross (‘Norstar’ source). The gamma ray induced *Ppd-A1*_del128c line was slightly earlier flowering than Paragon by two days (Fig. 2). All lines with the *Ppd-D1*_delN mutation flowered at the same time as ‘Paragon’, as did plants with the *Ppd-D1*_del143a gamma ray mutation. All lines with the gamma ray induced *Ppd-B1*_del211a or *Ppd-B1*_del319c mutations were significantly later flowering than ‘Paragon’ (11 to 14 days) (Fig. 2).

#### 2_IM genotypes

Families with the *Ppd-A1*_delCN and *Ppd-D1*_delN alleles flowered at the same time as families with the *Ppd-A1*_delCN alone, showing that the *Ppd-D1*_Mar and *Ppd-D1*_delN alleles did not significantly affect flowering when a functional *Ppd-B1* gene was present.

Combining the *Ppd-B1*_del211a or *Ppd-B1*_del319c deletions with the *Ppd-A1*_delCN allele gave an additional and highly significant delay in flowering of about 10 days over the *Ppd–B1* deletions alone. This was greater than the difference between ‘Paragon’ and *Ppd-A1*_delCN alone, showing that the *Ppd-A1*_delCN introgression had a stronger phenotypic effect when *Ppd-B1* was absent (Fig. 2).

Combining the *Ppd-B1*_del211a mutation with *Ppd-D1*_delN gave a small but significant delay in flowering of 3 days (P<0.001) over the single *Ppd-B1*_del211a introgression line. The combination of *Ppd-D1*_delN with *Ppd-B1*_del319c was not significantly later than the single *Ppd-B1*_del319c introgression line.

#### 3_IM genotypes

Triple mutant families combining the *Ppd-B1*_del211a or *Ppd-B1*_del319c deletions with *Ppd-A1*_delCN and *Ppd-D1*_delN had the latest flowering phenotype taking 28 and 30 days longer to flower in Experiment 1 than Paragon. This was significant for both *Ppd-B1* deletions. This suggested that *Ppd-D1*_Mar retains a low level of correct splicing and function and that this difference from the *Ppd-D1*_delN allele was detectable when *Ppd-A1* and *Ppd-B1* function were removed (Fig. 2).

### Flowering time of lines with candidate loss of function alleles_Experiment 2

In the second experiment ‘Paragon’ control plants were compared to lines with candidate loss of function alleles in a glasshouse with natural long days. The results were very similar to the fixed 18 h day experiment except that the difference in flowering times between ‘Paragon’ and the latest flowering lines was reduced by about 5 days (Fig. 2). This is probably because the plants in experiment 2 were grown under natural day lengths and did not experience day lengths sufficient to trigger photoperiod response in the early stages of development.

### Effect of *Ppd-1* mutations on spikelet number

Previous work on the effects of PI *Ppd-1a* alleles showed that their early flowering phenotype was associated with decreased spikelet number [5]. This suggests that the effect of the PI mutations is to compress the life cycle, reducing the time to the initiation of flower development and the time from spike initiation to the production of the terminal spikelet. The availability of late flowering genotypes allowed this effect to be tested over a greater range of flowering times.

**Figure 3:**
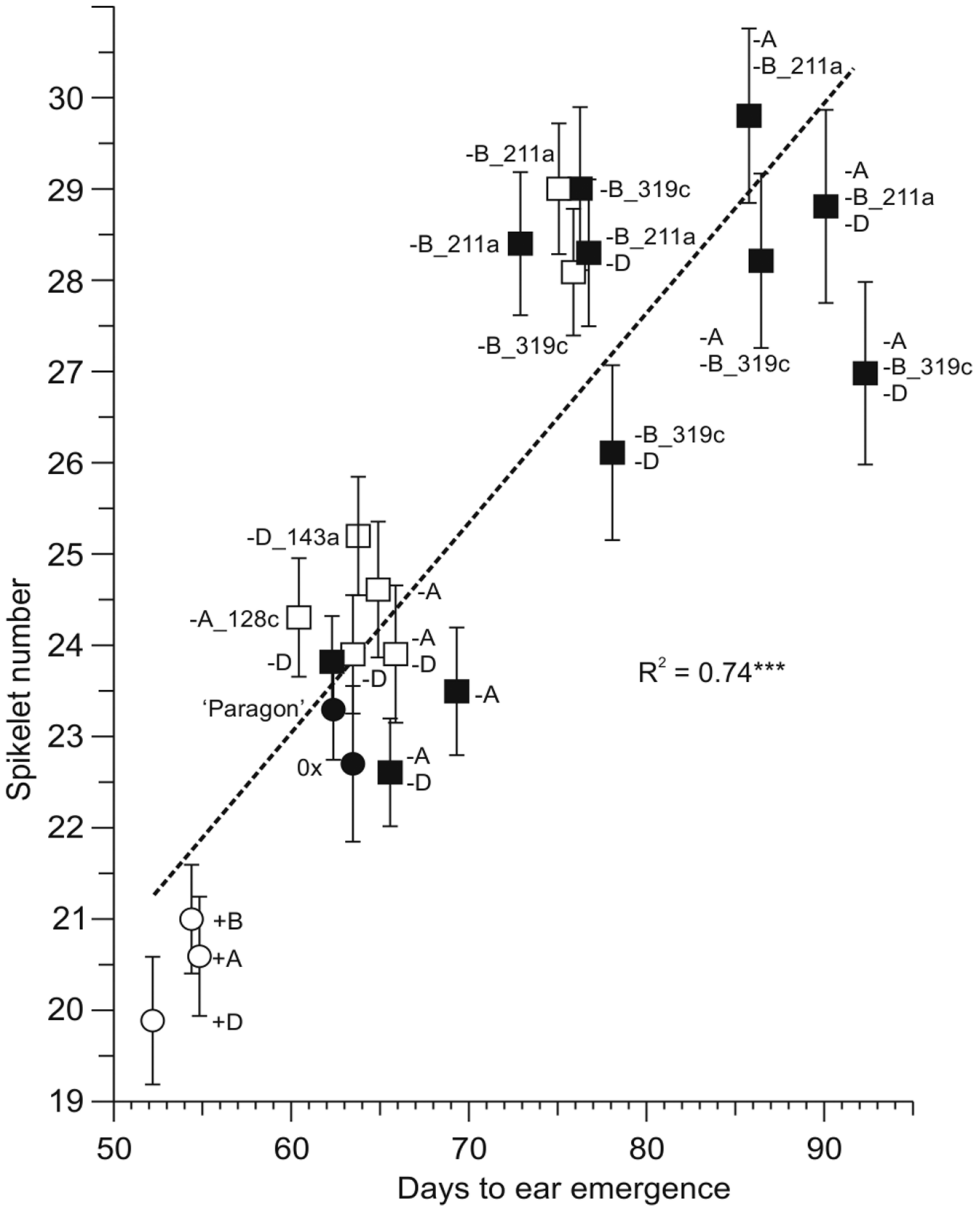
Correlation of spikelet number with days to ear emergence. Mean values of the ‘Paragon’ parent and introgression lines with photoperiod insensitive (*Ppd-1a*) or loss of function alleles of *Ppd-1* are plotted against flowering time (days to ear emergence on the main stem). Solid black circles show ‘Paragon’ and control (0×) means. White circles show introgression lines with a *Ppd-1a* (PI) allele on one genome (indicated by a+sign). Introgression lines with a loss of function allele on one or two genomes (indicated by a−sign) are shown by open squares. Lines derived from the 3× cross with a loss of function allele on one, two or three genomes are shown by solid squares. Error bars show standard deviations.

The spike on the main stem plus the leading tiller of each plant in Experiment_1 was analysed, counting each visible spikelet and the terminal spikelet irrespective of whether or not they had set seed. This showed a significant correlation between mean spikelet number and flowering time (R^2^ = 0.74). Plants with *Ppd-1a* (PI) alleles were earlier flowering than ‘Paragon’ and had two or three fewer spikelets. Spikelet number was similar to ‘Paragon’ for plants with *Ppd-A1*_delCN or *Ppd-D1*_delN single mutations or for plants with *Ppd-A1*_delCN+*Ppd-D1*_delN. Later flowering plants with the *Ppd-B1*_del211a or *Ppd-B1*n_del319c deletions, alone or in combination, had three to six additional spikelets, correlated with the degree of delayed flowering (Fig. 3).

**Figure 4:**
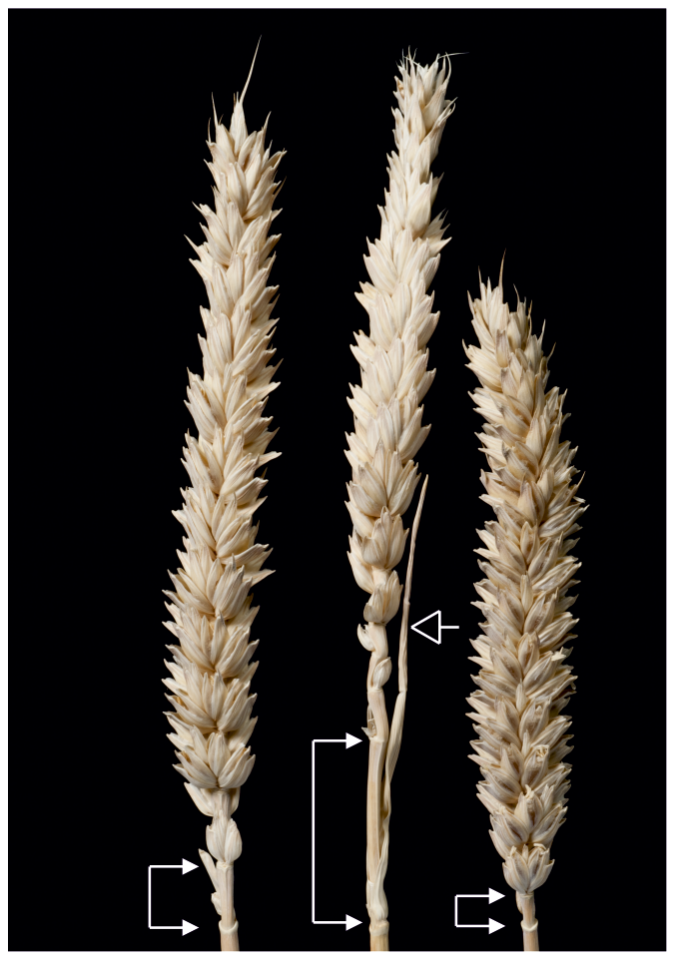
Spike phenotypes of 3_IM (*Ppd-A1*_delCN+*Ppd-B1*_del319c+*Ppd-D1*+delN) plants (left and centre) showing extended basal internode (solid arrowheads) and a leaf-like organ (open arrow) compared to a ‘Paragon’ control (right).

An additional feature of the latest flowering genotypes was an increase in internode length at the base of the spike and, in some plants, the appearance of a leaf like organ in place of a spikelet at the most basal spike internode (Fig. 4). This suggests that later flowering lengthened the phase of spike development, allowing more spikelets to form, and extended the transition period between vegetative and floral states so that some basal nodes had intermediate developmental characteristics.

### Gene expression over a 24 h period in introgression lines with loss of function alleles

**Figure 5:**
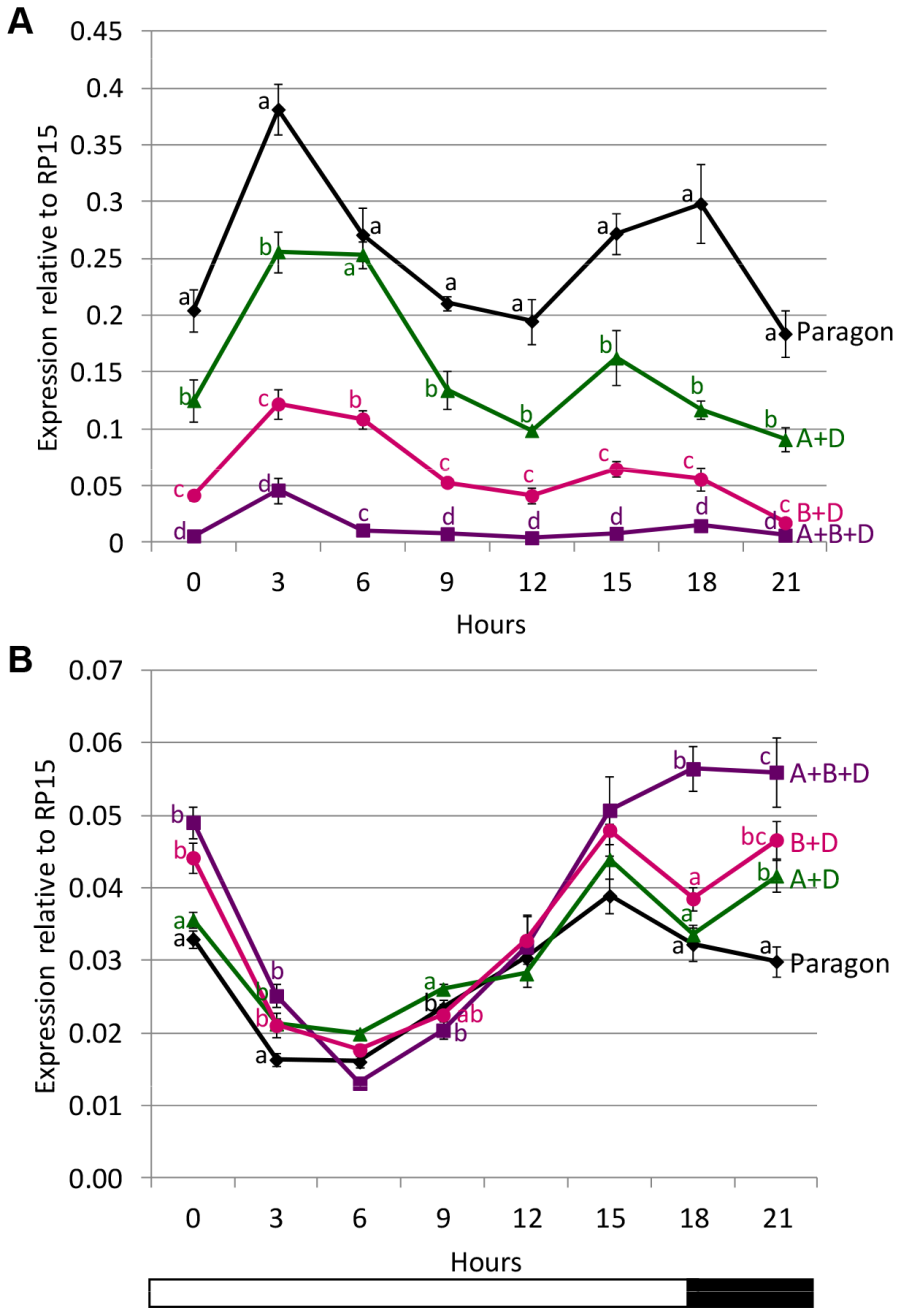
*TaFT1* (A) and *TaCO1* (B) expression over a 24 h period under long day (18 h light, 16°C) conditions. Plants were sampled every three hours with each point representing the mean and standard error of three biological replicates relative to *TaRP15*. Gene expression was analysed in the late flowering introgression lines *Ppd-A1*_delCN+*Ppd-D1*_delN (A+D) (green triangle), *Ppd-B1*_del319c+*Ppd-D1*_delN (B+D) (pink circle), *Ppd-A1*_delCN+*Ppd-B1*_del319c+*Ppd-D1*_delN (A+B+D) (purple square) and the control Paragon (black diamond). At each time point lines which share the same mean by t-test have the same letter.

The expression of *TaFT1* was analysed under long day conditions (18 h light) over a 24 h period using 22 day old plants (equivalent to plants in [13]) containing various combinations of *Ppd-1* loss of function alleles. Introgression lines were selected for varying delays in flowering time with *Ppd-A1*_delCN+*Ppd-D1*_delN having a small delay (2–5 days), *Ppd-B1*_del319c+*Ppd-D1*_delN having an intermediate delay (10–16 days) and *Ppd-A1*_delCN+*Ppd-B1*_del319c+*Ppd-D1*_delN having a large delay (20–30 days) compared to ‘Paragon’ controls (Fig. 2). It was hypothesised that levels of *TaFT1* would be reduced as elevated levels of *TaFT1* expression were previously found in early flowering introgression lines carrying *Ppd-1a* alleles [13]. The *TaFT1* circadian expression pattern was similar in all genotypes with a peak at three hours after dawn and a second peak at 15 to 18 h. However, *TaFT1* levels were significantly lower in lines with one or more introgressed candidate loss of function alleles and the decrease was proportional to the delay in flowering time so that the latest flowering genotype had the lowest expression (Fig. 5a).

The *CONSTANS* (*CO*) gene is a positive regulator of *FT* in Arabidopsis, but we previously showed that *TaCO1* levels are reduced in plants with *Ppd-1a* mutations and elevated *TaFT1* expression, possibly as the result of a feedback effect [13]. Consistent with this, peak *TaCO1* expression levels were significantly higher in plants carrying loss of function alleles of *Ppd-1* (Fig. 5b). This was related to the delay in flowering as *Ppd-A1*_delCN+*Ppd-B1*_del319c+*Ppd-D1*_delN had the highest level of *TaCO1* expression and took the longest time to flower (Fig. 2), while ‘Paragon’ took the shortest time to flower and had the lowest level of *TaCO1* expression. In ‘Paragon’, *TaCO1* expression peaked at 15 hours; in *Ppd-A1*_delCN+*Ppd-D1*_delN and *Ppd-B1*_del319c+*Ppd-D1*_delN, *TaCO1* expression peaked at 15 hours and again at 21 hours; while in *Ppd-A1*_delCN+*Ppd-B1*_del319c+*Ppd-D1*_delN expression peaked at 18 hours and remained high until dawn.

### Gene expression over a 6 week time course in introgression lines with loss of function alleles

This experiment included ‘Paragon’, the three introgression lines used for the 24 h time course experiment (above) plus the following four introgression lines: *Ppd-A1*_delCN, *Ppd-B1*_del319c, *Ppd-D1*_delN, *Ppd-A1*_delCN+*Ppd-B1*_del319c. The early flowering ‘Paragon (*Ppd-D1a*)’ line was also included for comparison. Lines were sampled 9 h after dawn each week over a time course of six weeks.

**Figure 6:**
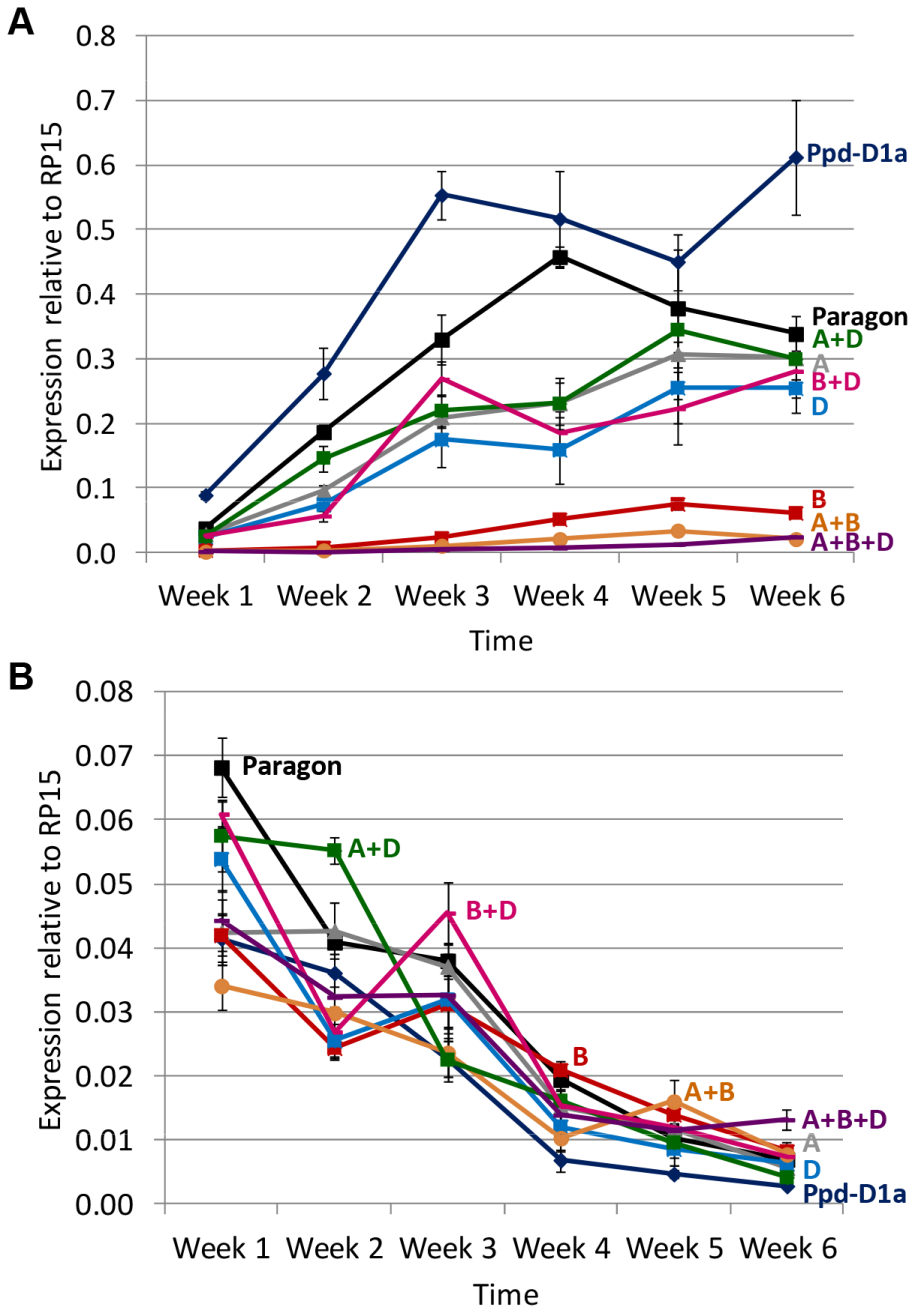
*TaFT1* (A) and *TaCO1* (B) expression over a six week time course. Plants were grown under long day conditions (18 h light; 16°C) and sampled 9 h after dawn at 7 day intervals. Each point shows the mean and standard error of three biological replicates relative to *TaRP15*. Gene expression was analysed in the late flowering introgression lines *Ppd-A1*_delCN (A) (grey triangle), *Ppd-B1*_del319c (B) (red square), *Ppd-D1*_delN (D) (blue square), *Ppd-A1*_delCN+*Ppd-B1*_del319c (A+B) (orange circle), *Ppd-A1*_delCN+*Ppd-D1*_delN (A+D) (green square), *Ppd-B1*_del319c+*Ppd-D1*_delN (B+D) (pink dash), *Ppd-A1*_delCN+*Ppd-B1*_del319c+*Ppd-D1*_delN (A+B+D) (purple dash), the control Paragon (black square) and the early flowering line ‘Paragon (*Ppd-D1a*)’ (dark blue diamond).

*TaFT1* expression increased gradually over the 6 week period in all genotypes and expression levels were generally proportional to flowering time (Fig. 6a). The early flowering *Ppd-D1a* introgression had higher expression than ‘Paragon’ controls, as shown previously [13] under short days, with a peak in expression at 3 weeks compared to 4 weeks in ‘Paragon’. Expression levels in the loss of function introgression lines were consistently lower than ‘Paragon’ and formed two groups with intermediate or very low *TaFT1* expression. Lines lacking *Ppd-B1* were consistently the latest flowering (Fig. 2) and comprised the very low expression group with the exception of *Ppd-B1*_del319c+*Ppd-D1*_delN which was late flowering but appeared in the intermediate expression group despite having low expression in the 24 h time course experiment (Fig. 5a). The intermediate expression group also contained the *Ppd-A1*_delCN, *Ppd-A1*_delCN+*Ppd-D1*_delN alleles and the *Ppd-D1*_delN genotype, the latter being very similar to ‘Paragon’ in flowering time. While *TaFT1* expression increased over the 6 week period, *TaCO1* expression steadily decreased (Fig. 6b). The *Ppd-D1a* genotype was consistently lower than ‘Paragon’, as shown previously [13], but a pattern in the loss of function genotypes was unclear. Sampling at an alternative time during the day might be required to reveal effects on *TaCO1* in the loss of function genotypes.

### *Ppd-A1*_delCN, *Ppd-D1a*, *Ppd-D1*_delN and *Ppd-D1*_Mar allele frequency in the Watkins and GEDIFLUX collections

The above results show that candidate loss of function alleles on the A and D genomes can affect flowering time, therefore, it is useful to have more information on their frequency and distribution as they were originally identified while sequencing *Ppd-1* genes from a small number of wheat genotypes with genetically defined photoperiod responses [7]. To gain information on allele frequency and distribution, and for comparison with [26], two contrasting germplasm collections were analyzed. We genotyped 769 accessions from the Watkins collection of land races and varieties collected in the 1920s and 1930s and 421 accessions from the GEDIFLUX collection of winter wheat varieties grown in the UK and North Western Europe between the 1940s and 1980s.

**Figure 7:**
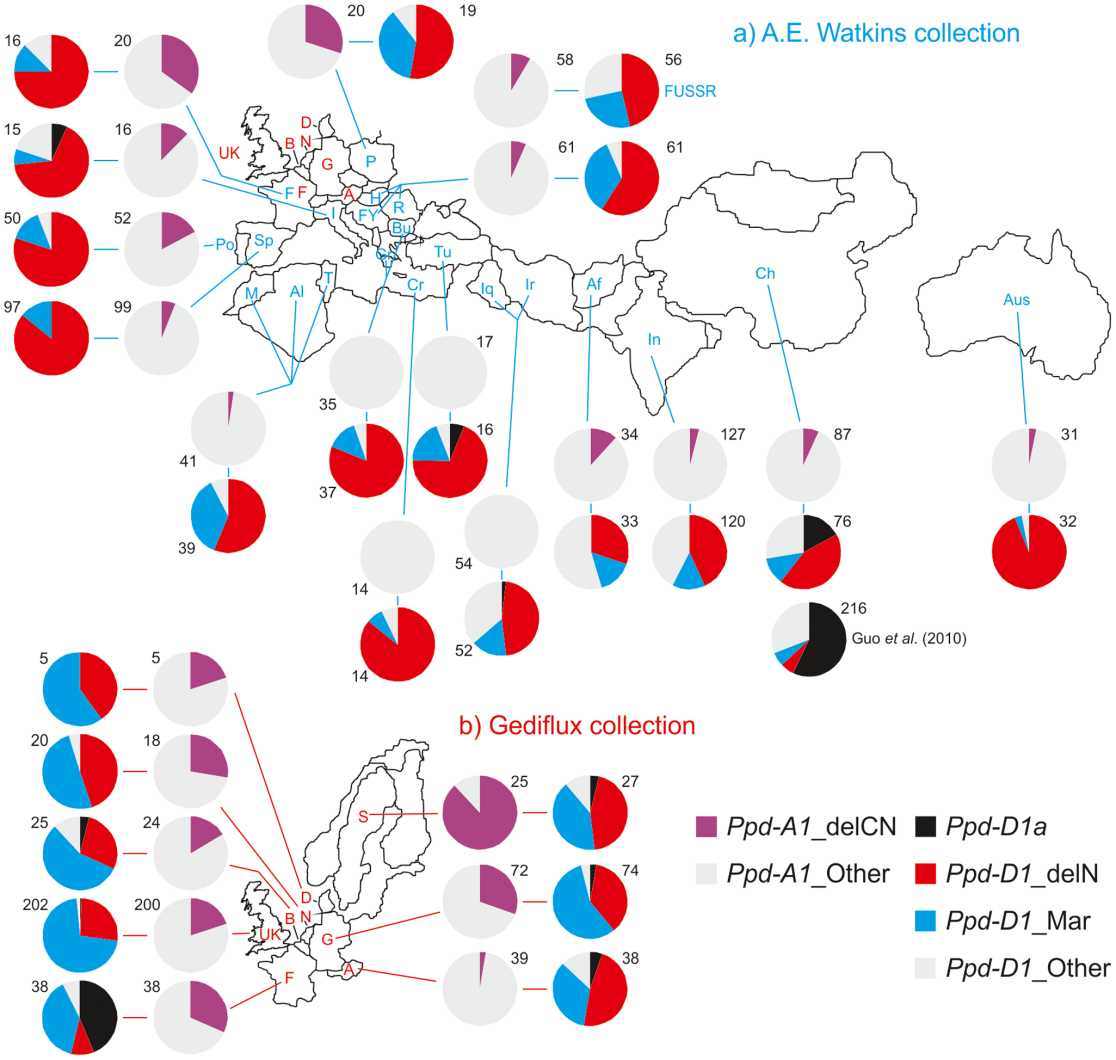
Frequency of *Ppd-1* mutant alleles in germplasm collections. a) A.E. Watkins (769 accessions), b) GEDIFLUX (421 accessions). Numbers by each pie chart are the number of accessions scored. Where the number of accessions per country in the Watkins collection was less than 12, adjacent countries were combined. Allele percentages are given in Table S2. The 216 modern Chinese accessions analyzed by Guo *et al.* (2010) are included in A) for comparison. Abbreviations represent Afghanistan (Af), Algeria (Al), Australia (Aus), Austria (A), Belgium (B), Bulgaria (Bu), China (Ch), Crete (Cr) Denmark (D), France (F), Germany (G), Greece (Gr), Hungary (H), Italy (I), India (In), Iraq (Iq). Iran (Ir), Morocco (M), Netherlands (N), Poland (P), Portugal (Po), Romania (R), Spain (Sp), Sweden (S), Turkey (Tu), Tunisia (T), former Union of Soviet Socialist Republics (FUSSR), United Kingdom (UK), former Yugoslavia (FY).

The *Ppd-D1a* (PI) allele was included as a control with a predicted distribution. It was widely adopted in the second half of the 20^th^ century and was expected to be absent from the Watkins collection or present mainly in material from the Far East [33], [34]. In the GEDIFLUX collection *Ppd-D1a* was expected to be common only in accessions from France, the most southerly country sampled, based on the known European distribution of this allele [32]. Results were consistent with expectations. Most Watkins accessions with *Ppd-D1a* were from China (Fig. 7, allele percentages are given in Table S2). This showed that the two collections are suitable for assessing allele distribution in relation to environment.

The *Ppd-A1*_delCN allele had an overall frequency of 7% in the Watkins collection. It was most common in France (35%) and Poland (30%) and was absent or rare in countries to the North-East or South-West of the Mediterranean, India, China and Australia. In the GEDIFLUX collection the overall frequency was 25% with a range from 3% in Austria to 88% in Sweden, with a significant correlation of frequency with latitude. This suggests this allele might be favoured in Northern environments.

The *Ppd-D1*_delN allele had an overall frequency of 60% in the Watkins accessions and was never less than 30% regionally. The overall frequency in the GEDIFLUX accessions was 30% and was only less than 25% in France. The *Ppd-D1*_Mar allele was found in 17% of the Watkins accessions and was more common in the GEDIFLUX accessions (58%). Both alleles were widely distributed. The combined frequency of the *Ppd-D1*_delN plus *Ppd-D1*_Mar alleles was high, falling below 65% in the Watkins accessions only in Afghanistan, India, Iran and Iraq. In the GEDIFLUX collection the combined frequency only fell below 80% in France where the *Ppd-D1a* allele was common. A Chi-square test of the GEDIFLUX collection showed that the proportion of accessions with *Ppd-D1*_delN or *Ppd-D1*_Mar plus *Ppd-A1*_delCN was as expected based on the frequency of the individual alleles (Table S3). This suggests that double mutant types have not been selected for or against.

## Discussion

In this study we investigated mutations in *Ppd-1* that are likely to result in loss of function or reduced function. Sequencing of cDNAs confirmed that *Ppd-A1*_delCN and *Ppd-D1*_delN alleles produce transcripts that are predicted to give proteins that have no function or severely compromised function. In ‘Paragon’ itself, the *Ppd-B1* gene provides about 90% of the *Ppd-1* transcript and the *Ppd-A1* and *Ppd-D1* genes have mutations that impair function [13]. The *Ppd-A1*_promdel allele is weakly expressed and the *Ppd-D1*_Mar allele produces predominantly mis-spliced transcript. From this we predicted that loss of *Ppd-B1* function would give a significant delay in flowering under long days analogous to the effect of the recessive *ppd-H1* mutation in barley [3].

### Introgression line phenotypes

Multiple introgression lines were developed in the spring wheat background ‘Paragon’. Gamma-ray induced deletion lines were chosen to ensure complete removal of the *Ppd-B1* gene. The predominant *Ppd-1* transcript in ‘Paragon’ is from the B genome [13] and deletion of this gene caused a highly significant delay in flowering by 10 to 15 days. This approach is open to the criticism that the late flowering phenotype might be due to the loss of adjacent genes, but the additional lateness of genotypes with combinations of *Ppd-1* mutations (Fig. 2) favours a direct effect of *Ppd-B1*. This could be confirmed using *Ppd-B1* mutations derived from TILLING populations but would not be possible in TILLING populations that have a duplication of the *Ppd-B1* gene. The *Ppd-A1*_delCN introgression had a small but significant delay in flowering compared to ‘Paragon’, while the significance of the *Ppd-D1*_delN and *Ppd-D1*_Mar mutations was less clear. *Ppd-D1*_delN had little or no effect when introduced into ‘Paragon’, suggesting that the *Ppd-D1*_delN and *Ppd-D1*_Mar alleles are phenotypically equivalent when a functional *Ppd-1* gene is present on one or more of the other genomes. However, the *Ppd-D1*_delN and *Ppd-D1*_Mar alleles could be distinguished in genotypes lacking *Ppd-A1* and *Ppd-B1*, possibly because the *Ppd-D1*_Mar retains a low level of correct splicing. The effect of the *Ppd-A1*_delCN mutation was also greater when combined with a *Ppd-B1* deletion. Therefore, the effect of *Ppd-A1*_delCN or *Ppd-D1*_delN could be more significant in genotypes where *Ppd-B1* is less active. It would therefore be of interest to explore wheat germplasm more widely to see if the predominant role of *Ppd-B1* observed in ‘Paragon’ is typical and to determine if variation in *Ppd-B1* expression or function exists. Varieties that are late flowering under long day conditions would be a logical starting point for this investigation. If the predominance of B genome transcript found in ‘Paragon’ is typical then loss of function mutations in this gene may have been selected against because significantly delayed development would be disadvantageous. In addition, *Ppd-B1* has been shown to vary in copy number [10], which would affect the ease with which loss of function alleles could arise. It may be possible to select *Ppd-B1* alleles with a range of activity from TILLING populations, but this would depend on the endogenous copy number.

### Flowering time is correlated with *TaFT1* expression

Introgression lines with different combinations of candidate loss of function mutations showed that progressively later flowering times under long days was associated with a parallel reduction in *TaFT1* expression (Fig. 5, 6). This is consistent with previous results where combinations of *Ppd-1a* (PI) alleles that gave progressively earlier flowering had associated increases of *TaFT1* expression [13]. Loss of function mutations of *Ppd-1* therefore delay flowering and extend life cycle length while *Ppd-1a* (PI) mutations accelerate flowering and reduce life cycle length. This is achieved, at least in part, by increasing or decreasing *TaFT1* expression, respectively.

In the *Ppd-1* loss of function genotypes lower *TaFT1* levels were associated with a higher peak in *TaCO1* levels (Fig. 5b). This inverse relationship between *TaFT1* expression and *TaCO1* expression is consistent with previous results from *Ppd-1a* genotypes and the hypothesis that there may be a feedback mechanism between *TaFT1* and *TaCO1* [13], [35]. No clear interaction was identified between *TaCO2* and *TaFT1* in the *Ppd-1a* genotypes [13]. Therefore, *TaCO2* was not analysed in this study.

Fig. 2 shows that candidate triple loss of function plants (*Ppd-A1*_delCN+*Ppd-B1*_del319c+*Ppd-D1*_delN) had the latest flowering time. However, flowering was still achieved and these plants expressed *TaFT1*, although at a much lower level (Fig. 5, 6). This suggests that some residual *Ppd-1* activity remains in these lines or, more probably, that *Ppd-1* is important but not essential for flowering. The existence of alternative routes to flowering is likely as several genetic pathways converge on *FT* in Arabidopsis or the *FT* equivalents *Hd3a* and *RFT1* in rice (*Oryza sativa*) to affect flowering time (reviewed in [36]). Wheat is likely to be similar. In Barley, *FT*-like genes have been suggested to function as floral activators under SD conditions [14], [37]. These *FT*-like genes may also provide an alternative route to promote flowering under LD's.

### Frequency of candidate loss of function alleles

Genotyping of the GEDIFLUX and Watkins collections showed that candidate loss of function alleles were common and widely distributed in modern NW European material and in older landrace material. However, given the small phenotypic effects observed in the introgression lines it is questionable whether this reflects any adaptive significance. However, we found weak evidence of geographical variation in the distribution of the *Ppd-A1*_delCN allele, with particularly high frequency in Sweden.

The combined frequency of *Ppd-D1*_delN and *Ppd-D1*_Mar was always high in the GEDIFLUX collection. Therefore, with the exception of accessions carrying the *Ppd-D1a* (PI) allele the GEDIFLUX wheat varieties have little or no *Ppd-D1* function. In the Watkins collection the combined frequency was also high except in the Middle East, Afghanistan, India and China, although, even here the frequency was usually over 50%. The lack of a clear phenotypic effect from loss of function or reduced function *Ppd-D1* mutations is consistent with results from [26] who found no clear association with environment. The reason for the prevalence of these alleles is therefore unclear. It is possible that analyzing distribution by country provides insufficient detail to see a relationship between allele and environments. Alternatively, the high frequency could be the result of founder effects or of historical selection for linked genes with favourable alleles for other traits.

To study this further, our preferred route is to assess the field performance of introgression lines in different environments. In addition, further potentially functional alleles of *Ppd-A1* and *Ppd-D1* have been identified by sequencing [7]. Introgressing these into ‘Paragon’ lines with loss of function alleles on the other genomes would provide a valuable way of assessing their properties. As ‘Paragon’ introgression lines already exist for a range of *Ppd-1a* (PI) alleles [10], [13], [27] it will be possible to combine photoperiod insensitive, wild type or loss of function alleles in multiple combinations. These can be used to assess effects on flowering time in detail.

## Supporting Information

Table S1 ***Ppd-1*** **genotypes of introgression lines and selections from the 3× cross.** Wild type alleles are in bold, candidate weak or reduced function alleles are underlined and candidate loss of function alleles are double underlined.
(DOCX)

Table S2 **Frequencies of** ***Ppd-1*** **mutations in the Watkins (W_) and GEDIFLUX (G_) collections.** * In the Watkins collection countries with accession numbers less than 12 were combined with geographical neighbours. The percentages are shown in graphic form in Figure 1.
(DOCX)

Table S3 **Observed and expected frequencies of single and double** ***Ppd-1*** **mutant combinations in the GEDIFLUX collection.** Expected frequencies are the product of the individual allele frequencies.
(DOCX)
