# Supplementary material for: Mutant Alleles of Photoperiod-1 in Wheat (Triticum aestivum L.) That Confer a Late Flowering Phenotype in Long Days
Source: PLoS One. 2013 Nov 14;8(11):e79459. doi: 10.1371/journal.pone.0079459 (PMC3828349; doi:10.1371/journal.pone.0079459)
Supplement: Table S1 — Ppd-1 genotypes of introgression lines and selections from the 3× cross. Wild type alleles are in bold, candidate weak or reduced function alleles are underlined and candidate loss of function alleles are double underlined. (DOCX) [file pone.0079459.s001.docx]

**Table S1: *Ppd-1* genotypes of introgression lines and selections from the 3× cross.** Wild type alleles are in bold, candidate weak or reduced function alleles are underlined and candidate loss of function alleles are double underlined.

|  | Ppd-A1 | Ppd-B1 | Ppd-D1 | Experiment 1  Plants (families) sampled | Experiment 2  Plants (families) sampled |
| --- | --- | --- | --- | --- | --- |
| ‘Paragon’ parent | *Ppd-A1*_promdel | ***Ppd-B1*** | *Ppd-D1_*Mar | 10 (1) | 10 (1) |
| Introgression of; |  |  |  |  |  |
| *Ppd-A1 ‘*Cappelle-Desprez’ source | *Ppd-A1*_delCN | ***Ppd-B1*** | *Ppd-D1_*Mar | 20 (4) | 20 (4) |
| *Ppd-A1* ‘Norstar’ source | *Ppd-A1*_delCN | ***Ppd-B1*** | *Ppd-D1*_Mar | 20 (4) | 19 (4) |
| *Ppd-A1* deletion | *Ppd-A1*_del128c | ***Ppd-B1*** | *Ppd-D1*_Mar | 10 (1) |  |
| *Ppd-B1* deletion | *Ppd-A1*_promdel | *Ppd-B1*_del211a | *Ppd-D1*_Mar | 20 (4) | 20 (4) |
| *Ppd-B1* deletion | *Ppd-A1*_promdel | *Ppd-B1*_del319c | *Ppd-D1*_Mar | 20 (4) | 20 (4) |
| *Ppd-D1* ‘Norstar’ source | *Ppd-A1*_promdel | ***Ppd-B1*** | *Ppd-D1*_delN | 21 (3) | 21 (3) |
| *Ppd-D1* deletion | *Ppd-A1*_promdel | ***Ppd-B1*** | *Ppd-D1*_del143a | 9 (1) |  |
| *Ppd-A1 + Ppd-D1* ‘Norstar’ source | *Ppd-A1*_delCN | ***Ppd-B1*** | *Ppd-D1*_delN | 15 (3) | 15 (3) |
| Selections from the 3× cross |  |  |  |  |  |
| 0_IM control (‘Paragon’ *Ppd* alleles) | *Ppd-A1*_promdel | ***Ppd-B1*** | *Ppd-D1*_Mar | 20 (4) |  |
| 1_IM *Ppd-A1* ‘Norstar’ source | *Ppd-A1*_delCN | ***Ppd-B1*** | *Ppd-D1*_Mar | 21 (3) | 14 (3) |
| 1_IM *Ppd-B1* deletion | *Ppd-A1*_promdel | *Ppd-B1*_del211a | *Ppd-D1*_Mar | 20 (1) | 19 (1) |
| 1_IM *Ppd-B1* deletion | *Ppd-A1*_promdel | *Ppd-B1*_del319c | *Ppd-D1*_Mar | 18 (3) | 21 (3) |
| 1_IM *Ppd-D1* ‘Norstar’ source | *Ppd-A1*_promdel | ***Ppd-B1*** | *Ppd-D1*_delN | 20 (4) | 20 (4) |
| 2_IM *Ppd-A1*+ *Ppd-B1* deletion | *Ppd-A1*_delCN | *Ppd-B1*_del211a | *Ppd-D1*_Mar | 20 (3) | 20 (3) |
| 2_IM *Ppd-A1*+ *Ppd-B1* deletion | *Ppd-A1*_delCN | *Ppd-B1*_del319c | *Ppd-D1*_Mar | 18 (4) | 20 (4) |
| 2_IM *Ppd-A1*+ *Ppd-D1* | *Ppd-A1*_delCN | ***Ppd-B1*** | *Ppd-D1*_delN | 18 (4) | 19 (4) |
| 2_IM *Ppd-B1* deletion + *Ppd-D1* | *Ppd-A1*_promdel | *Ppd-B1*_del211a | *Ppd-D1*_delN | 20 (3) | 21 (3) |
| 2_IM *Ppd-B1* deletion + *Ppd-D1* | *Ppd-A1*_promdel | *Ppd-B1*_del319c | *Ppd-D1*_delN | 14 (3) | 20 (4) |
| 3_IM *Ppd-A1* + *Ppd-B1* deletion + *Ppd-D1* | *Ppd-A1*_delCN | *Ppd-B1*_del211a | *Ppd-D1*_delN | 18 (1) | 17 (1) |
| 3_IM *Ppd-A1* + *Ppd-B1* deletion + *Ppd-D1* | *Ppd-A1*_delCN | *Ppd-B1*_del319c | *Ppd-D1*_delN | 18 (4) | 19 (4) |
