# Supplementary material for: Mutant Alleles of Photoperiod-1 in Wheat (Triticum aestivum L.) That Confer a Late Flowering Phenotype in Long Days
Source: PLoS One. 2013 Nov 14;8(11):e79459. doi: 10.1371/journal.pone.0079459 (PMC3828349; doi:10.1371/journal.pone.0079459)
Supplement: Table S2 — Frequencies of Ppd-1 mutations in the Watkins (W_) and GEDIFLUX (G_) collections. * In the Watkins collection countries with accession numbers less than 12 were combined with geographical neighbours. The percentages are shown in graphic form in Figure 1. (DOCX) [file pone.0079459.s002.docx]

**Table S2: Frequencies of *Ppd-1* mutations in the Watkins (W_) and GEDIFLUX (G_) collections.** * In the Watkins collection countries with accession numbers less than 12 were combined with geographical neighbours. The percentages are shown in graphic form in Figure 1.

|  | *Ppd-A1* | | *Ppd-D1* | | | |
| --- | --- | --- | --- | --- | --- | --- |
| Origin | n | *Ppd-A1*_delCN  Frequency (%) | n | *Ppd-D1a*_PI  Frequency (%) | *Ppd-D1*_delN  Frequency (%) | *Ppd-D1*_Mar  Frequency (%) |
| W_France | 20 | 7 (35.0) | 16 | 0 | 12 (75.0) | 2 (12.5) |
| W_Poland | 20 | 6 (30.0) | 19 | 0 | 10 (52.6) | 7 (36.8) |
| W_former USSR | 58 | 5 (8.6) | 56 | 0 | 26 (46.4) | 14 (25.0) |
| W_Portugal, Canary Islands* | 52 | 9 (17.3) | 50 | 0 | 40 (80.0) | 7 (14.0) |
| W_Spain | 99 | 6 (6.1) | 97 | 0 | 83 (85.6) | 14 (14.4) |
| W_Italy | 16 | 2 (12.5) | 15 | 1 (6.7) | 10 (66.7) | 1 (6.7) |
| W_Hungary*, Romania*, former Yugoslavia | 61 | 4 (6.6) | 61 | 0 | 36 (59.0) | 21 (34.4) |
| W_Greece, Bulgaria* | 35 | 0 | 37 | 0 | 30 (81.1) | 5 (13.5) |
| W_Turkey | 17 | 0 | 16 | 1 (6.3) | 11 (68.8) | 3 (18.8) |
| W_Crete | 14 | 0 | 14 | 0 | 12 (85.7) | 1 (7.1) |
| W_Morocca, Algeria*, Tunisia* | 41 | 1 (2.4) | 39 | 0 | 11 (56.4) | 14 (35.9) |
| W_Iran, Iraq* | 54 | 0 | 52 | 1 (1.9) | 24 (46.2) | 8 (15.4) |
| W_Afghanistan | 34 | 4 (11.8) | 33 | 0 | 10 (30.3) | 5 (15.2) |
| W_China | 87 | 6 (6.9) | 76 | 13 (17.1) | 33 (43.4) | 9 (11.8) |
| W_India | 127 | 5 (3.9) | 120 | 0 | 52 (43.3) | 17 (14.2) |
| W_Australia | 31 | 1 (3.2) | 32 | 0 | 30 (93.8) | 1 (3.1) |
| W_Total | 769 | 56 (7.3) | 737 | 16 (2.2) | 444 (60.2) | 129 (17.5) |
|  |  |  |  |  |  |  |
| G_Sweden | 25 | 22 (88.0) | 27 | 1 (3.7) | 12 (44.4) | 11 (40.7) |
| G_Denmark | 5 | 1 (20.0) | 5 | 0 | 2 (40.0) | 3 (60.0) |
| G_United Kingdom | 200 | 40 (20.0) | 202 | 1 (0.5) | 54 (26.7) | 144 (71.3) |
| G_Netherlands | 18 | 5 (27.8) | 20 | 0 | 9 (45.0) | 10 (50.0) |
| G_Germany | 72 | 22 (30.6) | 74 | 2 (2.7) | 27 (36.5) | 42 (56.8) |
| G_Belgium | 24 | 4 (16.7) | 25 | 1 (4.0) | 7 (28.0) | 14 (56.0) |
| G_Austria | 39 | 1 (2.6) | 38 | 2 (5.3) | 18 (47.4) | 13 (34.2) |
| G_France | 38 | 12 (31.6) | 41 | 18 (43.9) | 4 (9.8) | 16 (39.0) |
| G_Total | 421 | 107 (25.4) | 432 | 25 (5.8) | 133 (30.8) | 253 (58.6) |
