# Supplementary material for: Mutant Alleles of Photoperiod-1 in Wheat (Triticum aestivum L.) That Confer a Late Flowering Phenotype in Long Days
Source: PLoS One. 2013 Nov 14;8(11):e79459. doi: 10.1371/journal.pone.0079459 (PMC3828349; doi:10.1371/journal.pone.0079459)
Supplement: Table S3 — Observed and expected frequencies of single and double Ppd-1 mutant combinations in the GEDIFLUX collection. Expected frequencies are the product of the individual allele frequencies. (DOCX) [file pone.0079459.s003.docx]

**Table S3: Observed and expected frequencies of single and double *Ppd-1* mutant combinations in the GEDIFLUX collection.** Expected frequencies are the product of the individual allele frequencies.

|  | *Ppd-A1*_Other +  *Ppd-D1a* | *Ppd-A1*_Other +  *Ppd-D1*_delN | *Ppd-A1*_Other +  *Ppd-D1*_Mar | *Ppd-A1*_delCN +  *Ppd-D1a* | *Ppd-D1*_delN +  *Ppd-A1*_delCN | *Ppd-D1*_Mar +  *Ppd-A1*_delCN | Total * |
| --- | --- | --- | --- | --- | --- | --- | --- |
| Obs | 20 | 108 | 202 | 7 | 24 | 68 | 429 |
| Exp | 20.1 | 98.5 | 201.6 | 6.8 | 33.5 | 68.4 | 429 |

χ^2^ = 3.62; 5 d.f.; n.s.

* The total is greater than the 421 scored for *Ppd-A1* in Table S2 because this calculation includes a small number of genotypes with no country classification.
